# European exonerations: Factors and frequencies of wrongful convictions in Europe

**DOI:** 10.1016/j.fsisyn.2026.100710

**Published:** 2026-06-29

**Authors:** Teresa Schneider, Linda M. Geven, Jennifer M. Schell-Leugers

**Affiliations:** a Institute of Social Work and Law, Lucerne School of Social Work, Lucerne University of Applied Sciences and Arts, Lucerne, Switzerland; b Méndez Centre for Effective and Fair Interviewing (ZEFAB), Freiburg i.Br., Germany; c Institute for Criminal Law and Criminology, Faculty of Law, Leiden University, Leiden, the Netherlands; d University College Maastricht, Faculty of Science and Engineering, Maastricht University, Maastricht, the Netherlands

## Abstract

Wrongful convictions represent a profound failure of criminal justice systems with severe individual and societal consequences such as, undermining public trust in the justice system, allowing true perpetrators to remain free, and inflicting lasting harm on exonerees and their families. Despite growing international attention to this issue, systematic empirical data on wrongful convictions in Europe remains limited. This article presents a descriptive analysis of the 144 exoneration cases documented in the European Registry of Exonerations (EUREX) as of May 2026. The dataset includes exoneration cases from across Europe, with Germany, Italy, the Netherlands, Sweden, and Spain most represented. Findings show that the majority of exonerees were male, with most initial convictions relating to homicide. On average, individuals spent around 6 years and 5 months wrongfully imprisoned, collectively representing 927 lost years, while the average time from conviction to exoneration was nearly 12 years. False confessions, and false accusation or perjury emerged as the leading contributing factors, frequently occurring in combination with other factors. DNA evidence played a role in only a minority of exonerations These findings are consistent with North American findings and underscore the need for structured case review mechanisms and continued cross-national research into the causes and prevention of wrongful convictions.

## 1 Introduction

The number of confirmed wrongful convictions has increased substantially over the past decades [[1], [2], [3]]. Despite this growing recognition, it was still unclear how frequently and for what reasons wrongful convictions occur in European countries [4,5], since no official database existed to provide information on exoneration cases. This gap stands in stark contrast to developments in other jurisdictions, where systematic documentation efforts have significantly advanced the field. For example, in the United States, the Innocence Project^1^ has helped free or exonerate more than 250 wrongfully convicted individuals since 1992, primarily through post-conviction DNA testing. By 2020, it had compiled data on approximately 375 DNA exonerations and reported summary statistics for these cases. In addition, the National Registry of Exonerations (NRE),^2^ established in 2012, systematically documents a broader set of exoneration cases in the United States and currently includes more than 3800 cases (retrieved on April 22, 2026). Similar efforts have emerged elsewhere, including the Evidence-Based Justice Lab^3^ in the United Kingdom, which has documented 507 cases, and the Canadian Registry of Wrongful Convictions,^4^ established in 2023, which contains 93 cases (both retrieved on April 22, 2026). Together, these databases enable systematic investigation of wrongful convictions and their underlying factors. No equivalent infrastructure has existed for Europe as a whole, nor for most individual European countries. While the Evidence-Based Justice Lab provides a national registry for the United Kingdom, it does not offer a cross-national European perspective and is not included in EUREX. As a result, it remains unclear how often wrongful convictions occur across continental European jurisdictions, what factors drive them, and what their individual and societal consequences are.

To address this gap, the European Registry of Exonerations (EUREX)^5^ was established in 2024. EUREX aims to build a comprehensive, publicly accessible database of European wrongful conviction cases, providing academics, legal professionals, law enforcement, policymakers, and the public with systematic evidence on the number, causes, and consequences of wrongful convictions across Europe. This article describes EUREX's definitional framework, goals, and data collection procedures, and presents findings from the first 144 cases collected as of May 2026. Limitations and ongoing challenges are also discussed.

### 1.1 Definition

Terms related to wrongful convictions are frequently used interchangeably in the literature, despite important conceptual differences between them. Recent research has highlighted this conceptual ambiguity, showing that terms such as *wrongful conviction*, *miscarriage of justice*, *innocence*, and *exoneration* are often inconsistently defined—or not defined at all—with “wrongful conviction” and “exoneration” regularly used interchangeably [6]. In line with these concerns, we adopt a more precise terminology in this article. Specifically, we use *wrongful conviction* to refer to definitive convictions that have been retried and subsequently overturned, and we refer to the affected individuals as *exonerees*. For broader categories of cases or registries that do not meet this stricter definition, we use the term *miscarriage of justice*.

In line with the definitional framework adopted in the NRE, we include only wrongful conviction cases in which an individual was convicted of a crime by final judgment and was subsequently cleared through a post-conviction legal re-examination, for example following the emergence of new evidence (cf. [7]). This includes cases in which the crime was later attributed to another person or where it was established that no crime had occurred. A final conviction refers to a conviction that has become legally binding after all ordinary appeals have been exhausted or the time for appeal has expired. Consistent with this framework, EUREX excludes cases in which a conviction was overturned through direct appeal before becoming final. However, victims of such miscarriages of justice often suffer the same consequences as exonerees (e.g., prolonged detention, financial loss). One example is the prominent case of Amanda Knox, who spent almost four years in an Italian prison before being acquitted on appeal before a final conviction was established.

In addition, EUREX does not include cases in which individuals were found not criminally responsible due to mental illness, or cases in which a person was involved in the offence but to a lesser degree than originally determined. While such cases may also fall under broader notions of miscarriages of justice—and are particularly relevant in the European context (e.g., [8,9])—they fall outside the scope of the present definition. These definitional choices reflect deliberate boundaries established within the EUREX framework and ensure conceptual clarity and cross-national comparability. At the same time, it is important to recognize that the cases included in EUREX represent only a subset of all wrongful convictions and miscarriages of justice.

### 1.2 Consequences of wrongful convictions

Wrongful convictions have profound and far-reaching consequences for individuals, families, and society. For exonerees, harm extends beyond incarceration and includes loss of autonomy, social ties, and financial stability, as well as enduring psychological effects such as anxiety, depression, and post-traumatic stress disorder [[10], [11], [12]]. Beyond diagnosable conditions, wrongful imprisonment inflicts profound damage to identity and self-concept, with feelings of alienation and helplessness commonly persisting long after release [10]. The effects extend to families, who experience secondary victimization in the form of emotional distress, financial strain, and social stigma [13]. After release, exonerees frequently encounter continued stigma and structural barriers in employment, housing, and access to mental health care [14]. At the societal level, wrongful convictions undermine trust in the justice system and allow actual perpetrators to remain free [15]. In sum, these consequences highlight the importance of understanding the causes of wrongful convictions in order to better identify how they can be prevented.

### 1.3 Contributing factors of wrongful convictions

When analysing cases of wrongful convictions and exonerations, five main contributing factors emerge across various jurisdictions and registries: False confessions, false or misleading forensic evidence, flawed eyewitness identification (procedure), perjury or false accusation, and official misconduct [7]. While this typology is widely cited, alternative frameworks have also been proposed. For example, a six-factor model additionally includes inadequate legal defence (Leo, 2017). In the present study, we focus on the five factors identified by Gross and Shaffer [7], which align with the factors currently tracked in EUREX. Inadequate legal defence was not included as a coded factor due to limitations in the available data rather than its substantive importance. As prior research has shown, deficiencies in defence representation are often difficult to detect in case records, as they typically involve omissions (e.g., failure to investigate or challenge evidence) that leave little trace unless they are explicitly examined in post-conviction proceedings [7]. Given that EUREX relies largely on publicly available sources rather than full case files, it is not possible to assess this factor in a reliable and consistent manner across cases. Understanding these contributing factors is essential to identifying vulnerabilities in the criminal justice system and implementing reforms to prevent future errors.

#### 1.3.1 False confessions

False confessions are statements made by individuals admitting to crimes they did not commit. While voluntary false confessions occur without any pressure from the police and often to protect the real perpetrator [16], coerced false confessions happen due to pressure in the police interrogation to escape the situation [17,18]. When looking at exoneration cases, coerced false confessions are overrepresented compared to voluntary false confessions [7]. This is likely because voluntary false confessions are often uncovered before the conviction or because such confessors are not motivated to uncover the injustice because sanctions are relatively mild, or because they still want to protect the real perpetrator [19,20].

While a person in police custody is inherently vulnerable, research identified several individual risk factors for coerced false confessions. Youth, intellectual disability, and mental illness are overrepresented in cases of wrongful convictions due to false confessions [7,21,22]. In addition, personality traits such as suggestibility and compliance have been linked to coerced false confessions (for an overview see [23]). Beyond individual characteristics, several situational risk factors significantly increase the risk of false confessions. The context of the interrogation itself plays a crucial role: the longer and more frequently an individual is interrogated, the harder it becomes to resist increasing pressure, thereby raising the likelihood of confessing falsely simply to end the interrogation. Interrogation tactics that downplay the seriousness of the offence and the consequences of confession (minimization) or highlight the severe consequences of continued denial (maximization) can further lead innocent suspects to view a confession as their only option [[24], [25], [26], [27], [28]].

Moreover, conditions of detention themselves contribute to vulnerability. Being held in police custody is inherently stressful, particularly for innocent suspects. Individuals in pretrial detention are isolated from the outside world and may experience disrupted sleep and eating patterns. Such conditions can heighten susceptibility to pressure and suggestion [29], partly because cognitive capacities such as concentration and memory become impaired under fatigue [30,31]. Taken together, false confessions often arise from the interaction of individual vulnerabilities and situational pressures.

#### 1.3.2 False or misleading forensic evidence

Forensic evidence is routinely used in criminal proceedings to establish a physical connection between a suspect and a crime. This encompasses, for example, DNA analysis, fingerprint comparisons, bloodstain pattern analysis, hair and fibre examination, and a wide range of other trace evidence techniques. Despite its perceived objectivity, the evaluation of forensic evidence is associated with a substantial risk of error. For example, in the United States, Morgan [32] documented false or misleading forensic evidence in over 730 cases from the NRE, and the Innocence Project has identified invalidated or misapplied forensic science as a contributing factor in about half of the wrongful convictions in their database.^6^

Several distinct factors account for the contribution of forensic evidence to wrongful convictions. The first concerns the use of unvalidated or scientifically unsound techniques, so-called “junk science”. With the advent of DNA technology, numerous forensic disciplines previously accepted by courts (e.g., microscopic hair analysis, bite mark comparison) have been substantially discredited due to the absence of empirical validation for their foundational assumptions [33,34]. Beyond the use of unvalidated methods, even scientifically sound techniques are vulnerable to human error at the interpretive stage. Forensic judgments can be distorted by psychological biases, most notably contextual and confirmation bias [[35], [36], [37]]. For example, research by Dror et al. [38] demonstrated that fingerprint experts reached different conclusions when exposed to contextual information about a suspect's guilt. Common failures also include misreading laboratory results, overstating the probative value of findings, and over-interpreting ambiguous evidence [[39], [40], [41]]. At the more serious end of the spectrum lies presenting contradicted evidence, where conclusions are later rebutted by other experts, authoritative bodies, or post-conviction testing, and perjury or fraud, where forensic actors deliberately falsify or misrepresent results. The latter may also include concealing exculpatory findings from the defence, lying about professional credentials, or altering testimony in response to external influence. Together, these vulnerabilities underscore the need for standardised, double-blind procedures in forensic practice [32,42,43] and highlight why forensic evidence, despite its scientific appeal, cannot be treated as immune to misinterpretation.

#### 1.3.3 Flawed eyewitness identification (procedure)

Eyewitness misidentification is one of the most extensively studied contributing factor to wrongful conviction. In approximately 69% of the exoneration cases documented by the Innocence Project, eyewitness misidentification led to the original conviction.^7^ The literature distinguishes between three broad categories of factors affecting eyewitness reliability, which differ in the extent to which they can be influenced by investigative authorities.

First, estimator variables are outside the control of the justice system and include factors such as lighting conditions, viewing distance, or cross-race identification, [44]. In contrast, system variables are under the control of law enforcement and include lineup composition, administrator instructions, and the timing of identification procedures [45]. Research consistently shows that non-blind lineup administration, suggestive instructions, biased foil selection, and failure to record witness confidence at the time of identification all increase the risk of false identification [46]. Recently, Mickes and Wixted [47] have argued that wrongful convictions involving eyewitness error may arise less from inherent weaknesses in memory than from systemic failures to preserve and prioritise the witness's initial identification response, the least contaminated version, over subsequent, more rehearsed accounts.

A third category concerns how memory can be distorted by exposure to information encountered after the witnessed event. This so-called “misinformation effect” refers to the process whereby details from post-event sources, such as media reports, conversations with other witnesses, or police questioning, become incorporated into the original memory trace, potentially altering or displacing accurate recollections [48]. Contemporary research has extended this concern to digital contexts, demonstrating that exposure to social media content featuring suspects prior to a lineup can contaminate eyewitness memory in ways that increase the risk of false identification [49].

#### 1.3.4 Perjury or false accusation

Testimonial evidence from parties connected to an offence, including victims, eyewitnesses, and fellow inmates, carries substantial weight in criminal proceedings. However, such testimony can lead to wrongful convictions in two distinct ways: through (a) intentional false accusations or (b) unintentional false accusations due to memory failures. Intentional false accusations can occur due to many reasons, including financial gain, securing favourable plea agreements, deflecting suspicion, or seeking revenge [50,51]. Perjury represents a particularly serious form of intentional false accusation, occurring when a person (e.g., witness, law enforcement officer) under oath provides testimony they know to be partially or wholly untrue [52]. In the United States, perjury or false accusation was present in over half of all exonerations listed by the NRE, and in as many as 72% of exonerations recorded in 2024 alone. Not all false accusations, however, are deliberate. False memories, which are recollections of events that an individual genuinely believes to be accurate but that are objectively false, represent a second, unintentional pathway to wrongful accusation. Such memories can arise in both children and adults in response to suggestive interviewing techniques or therapeutic interventions [53,54]. Real-life instances of false memories frequently relate to memories and accounts of childhood sexual abuse (e.g., Worms Trial and Montessori Trial, Germany).

#### 1.3.5 Official misconduct

Official misconduct refers to cases in which police, prosecutors, or other government officials have abused their authority or manipulated the judicial process in ways that contributed to a wrongful conviction. This includes, for example, perjury by law enforcement or forensic experts, fabricating or misrepresenting evidence supporting the prosecution, withholding exculpatory evidence, tampering with witnesses, and misconduct during suspect interviews (e.g., [threats of] violence, drug administration). It commonly co-occurs with other contributing factors — particularly false accusation or perjury [55]. An analysis of the first 2.400 cases in the NRE shows that official misconduct contributed to the wrongful convictions of 54% of individuals who were later exonerated. The most common form of misconduct was the concealment of exculpatory evidence [56].

## 2 The current case analysis

The present article draws on data from EUREX to provide a first systematic empirical overview of wrongful convictions across Europe. We report descriptive findings on the geographic distribution of cases, the types of crimes involved, the duration of wrongful imprisonment, and the prevalence of key contributing factors. Together, these findings offer a preliminary empirical foundation for understanding patterns of wrongful convictions across Europe and provide a starting point for future comparative and policy-oriented research.

### 2.1 Case collection and coding procedure

In contrast to the United States, where court records are often publicly accessible under the principle of “open courts” and can frequently be searched through centralized online systems, European countries generally adopt a more restrictive approach. Across Europe, greater emphasis is placed on data protection and privacy rights, which limits public access to complete court files [57]. Therefore, data included in EUREX are collected exclusively from publicly available sources. Given cross-national differences in access to case materials, EUREX does not impose a uniform documentary requirement for case inclusion: cases may be identified based on different types and amounts of sources depending on availability across jurisdictions. Sources included newspaper articles, academic publications, book chapters, documentaries, podcasts, and publicly available court decisions. In several cases, additional information was obtained through direct contact with defence lawyers, local innocence organizations, or individuals with direct knowledge of the case. Whenever possible, we cross-checked multiple sources. In cases of conflicting information, priority was given to judicial decisions or detailed legal reporting. Ambiguities were discussed within the research team to arrive at consistent coding decisions. Each case was reviewed by members of the research team to determine eligibility based on the available information. The availability and level of detail of case information varied substantially both across cases and across jurisdictions. The registry is therefore a continuously developing dataset, with new cases actively sought and added as they become publicly identifiable.

**Fig. 1:**
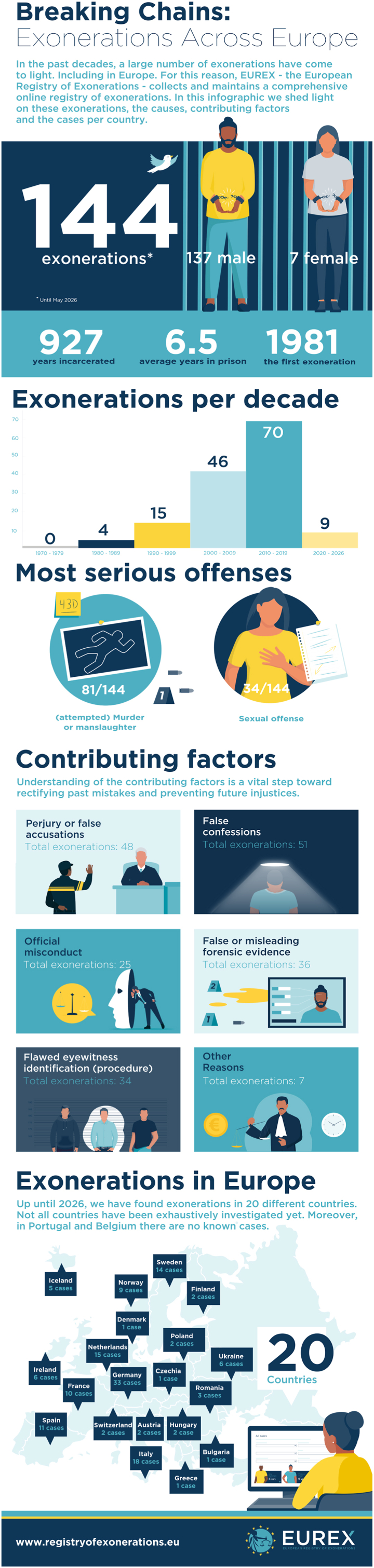
Overview of the EUREX data, including gender distribution, total and average years of imprisonment, exonerations by decade, most serious offences, contributing factors, and countries represented in the dataset.

For each case, information was coded across variables covering demographic characteristics, conviction details, contributing factors, and exoneration outcomes. Coding categories were informed by prior exoneration research and adapted to the European legal context. Cases were coded for one or more factors contributing to the wrongful conviction, including false confessions, flawed eyewitness identification (procedures), false or misleading forensic evidence, perjury or false accusation and official misconduct. Contributing factors were not treated as mutually exclusive. A factor was coded as present whenever it could be reasonably inferred from public sources, irrespective of whether it was explicitly acknowledged by a reviewing court. Cases were also coded according to the most serious offence of conviction and classified as involving either a real crime or no crime. No-crime cases were defined as cases in which it was established, whether through subsequent investigation or legal re-examination, that no criminal act had occurred. This also includes cases where an act initially prosecuted as a crime was later found to be accidental or otherwise non-criminal in nature. Age variables were coded only when numerical information was available. Entries coded as “adult” or missing were treated as missing in quantitative summaries. Temporal variables included year of conviction, year of exoneration, years incarcerated, and time between conviction and exoneration, and were coded only when sufficient information was available (see below for missing data by variable). Fig. 1 provides an overview of the data.

### 2.2 Demographic characteristics of exonerees

The overwhelming majority of exonerees were male (*n* = 137, 95.1%), while female exonerees represented a small minority (*n* = 7, 4.9%). Where numerical data were available, exonerees were on average around 32 years old (*n* = 111) at the time of the alleged offence, around 36 years old (*n* = 113) at conviction, and around 47 years old (*n* = 113) at exoneration, illustrating the extended time span many individuals spent under the shadow of a wrongful conviction. Five cases involved posthumous exonerations, underscoring that for some individuals, legal recognition of innocence occurred only after death.

### 2.3 Geographic and temporal distribution

The cases currently included in EUREX originate from 20 European countries, although their distribution is uneven. Germany accounts for the largest proportion of documented exonerations (*n* = 33, 23.1%), followed by Italy (*n* = 18, 12.6%), the Netherlands (*n* = 15, 10.5%), and Sweden (*n* = 14, 9.8%). The other 16 countries are represented by smaller numbers of cases. Despite systematic searches, no publicly documented exonerations since 1970^8^ were identified in Portugal, Belgium and Croatia. Several European countries, including Serbia, Lithuania, and Turkey, have not yet been reviewed in sufficient detail to allow inclusion. The first exoneration in the registry occurred in 1981, with the most recent in 2024. Most wrongful convictions at the original trial, later overturned, occurred in the 1990s (47 cases, 32.6%) and the 2000s (58 cases, 40.6%). By contrast, the formal recognition of innocence through post-conviction exoneration proceedings occurred predominantly in later periods, with nearly half of all exonerations issued in the 2010s (*n* = 70, 49.0%), followed by the 2000s (*n* = 46, 32.2%). This temporal divergence reflects the substantial time lag between conviction and exoneration observed across cases, with a mean interval of 11.8 years between the initial conviction at first instance and the formal exoneration. The longest documented interval was 42 years, in the case of Martin Conmey in Ireland.

### 2.4 Offence characteristics and crime classification

Wrongful convictions documented in EUREX predominantly concern serious criminal offences. More than half of all cases involved (attempted) murder or manslaughter (*n* = 81, 56.3%). Sexual offences formed the second largest category (*n* = 34, 23.6%). Less frequent categories included other offences (*n* = 12, 8.3%), violent offences not classified as homicide (*n* = 9, 6.3%), robbery or burglary (*n* = 7, 4.9%), and arson (*n* = 1, 0.7%). In 20 cases (13.9%), exonerees had been convicted of more than one offence, typically in combinations involving sexual offences or property-related crimes.

Based on publicly available sources, we were able to classify 130 exoneration cases according to whether a crime had in fact occurred. In the remaining cases, classification was not possible due to insufficient information. These cases primarily involved situations in which the alleged crime concerned a missing person, and it remained unclear from available sources whether a criminal act had occurred or whether the disappearance had non-criminal explanations (e.g., voluntary disappearance or accident). While most cases involved an actual criminal act (*n* = 91), a substantial subgroup consisted of no-crime cases (*n* = 39).

### 2.5 Wrongful incarceration and time to exoneration

Across all cases, exonerees spent a combined total of 927 years incarcerated, either in prison or in a mental health facility. The average length of wrongful incarceration was 6.4 years (*SD* = 5.7; *Mdn* = 5), with durations ranging from zero years (in cases without custodial sentences) to 31 years in the case of Dirk K., the longest period documented in EUREX. The largest proportion of exonerees served between 1 and 5 years (*n* = 61, 42.4%), followed by 6 to 10 years (*n* = 38, 26.4%). Smaller but notable groups served 11 to 15 years (*n* = 15, 10.4%) or were incarcerated for 16 years or more (*n* = 12, 8.3%). Within this latter group, four individuals (2.8%) were wrongfully incarcerated for 21 years or longer.

Particularly long intervals between conviction and exoneration were disproportionately observed in cases involving serious violent crimes and in cases where no alternative perpetrator was identified. Among the 12 cases involving 16 years or more of wrongful incarceration, several belonged to the group of unresolved crime cases, further illustrating how wrongful convictions may persist for decades once cases become effectively dormant.

### 2.6 Case review procedures and institutional involvement

In most cases (*n* = 132, 91.7%), exonerations occurred without the involvement of a formal case review unit. A dedicated review body contributed to only 12 cases (8.3%), reflecting considerable cross-national variation in the institutionalization of post-conviction review mechanisms. These findings suggest that in many European jurisdictions the correction of wrongful convictions still relies primarily on case-specific legal efforts rather than institutionalized review mechanisms.

### 2.7 Contributing factors to wrongful convictions

The most frequently identified factor was false confession, present in 51 cases (35.4%). This was followed by false accusation or perjury (*n* = 48, 33.3%), false or misleading forensic evidence (*n* = 36, 25.2%), and flawed eyewitness identification (procedures) (*n* = 34, 23.6%). Official misconduct was identified in 25 cases (17.4%) and 7 cases (4.7%) involved other reasons. In 49 cases (34.0%), more than one contributing factor was identified. This illustrates that wrongful convictions frequently emerge from cumulative evidentiary and procedural failures, rather than from a single point of error within the criminal justice process.

### 2.8 DNA evidence and identification of the real perpetrator

Among the 91 cases in which a crime had occurred, 20 (22.0%) involved DNA exonerations; the others were overturned following re-evaluation of witness testimony, forensic evidence, or procedural errors. In 54 of the cases in which a crime had occurred, a real perpetrator was identified (59.3%), whereas in 36 cases innocence was established without definitive identification of an alternative offender (40%; one case lacked information).

## 3 Discussion

This article presents the first descriptive overview of 144 wrongful conviction cases documented in EUREX as of May 2026. The results provide empirical insight into patterns of wrongful convictions across European jurisdictions, which have previously been under-documented in the literature. Even though this database does not include all wrongful conviction cases across Europe and the true prevalence of such convictions is unknown, the findings contribute to a more systematic understanding of the characteristics and contributing factors of wrongful convictions across European jurisdictions.

### 3.1 Upward trend in the numbers of recorded exonerations

The present data revealed a clear upward trend in the number of recorded exonerations over time, with the 2010s accounting for the highest number of cases (*n* = 70, 49.0%), followed by the 2000s (*n* = 46, 32.2%), and relatively few cases in the decades prior. This pattern is consistent with trends documented in international literature and can be attributed to several interrelated developments. First, advances in DNA technology since the late 1980s have played a key role in exonerating innocent people. As a result, awareness and acceptance of the fact that innocent people can be wrongfully convicted has grown significantly over the past two decades [58]. However, when looking at the EUREX cases, DNA contributed to only about 22% (*n* = 20) of exonerations when a crime had occurred suggesting that the rise in exonerations cannot be attributed to DNA alone. Second, the growth of the innocence movement has been instrumental in uncovering wrongful convictions. The establishment of innocence projects and conviction review organizations from the 1990s onward created a dedicated legal and advocacy infrastructure for post-conviction review that had not previously existed. This institutional development is likely reflected in the increase in European exonerations from the 2000s onward. Third, the increase may partly reflect a growing willingness within legal systems to acknowledge and correct wrongful convictions driven by increased public and political attention to the issue. However, the rise in recorded exonerations over time may partly reflect improvements in documentation and registry coverage rather than a true increase in the rate at which wrongful convictions occur [59]. Cases from earlier decades may be underrepresented simply because systematic recording did not yet exist and even if it had, awareness of such cases and access to case files may be restricted. Furthermore, most registries establish a specific starting point for data collection. For example, EUREX includes European exoneration cases only if the original conviction occurred in 1970 or later, whereas the NRE covers exoneration cases from 1989 onwards.

### 3.2 Innocent individuals wait around 12 Years to be exonerated in Europe

In the present dataset, the mean time from conviction to exoneration was 11.8 years, with a range of 1 to 42 years. This prolonged delay has profound implications for the individuals concerned. Exonerees in this sample were on average 47 years old at the time of exoneration, meaning that a substantial portion of their adult lives had elapsed before their innocence was formally recognised. The length of wrongful incarceration itself is equally striking: exonerees served an average of 6.4 years in prison, with some individuals incarcerated for over three decades. These figures underscore that wrongful conviction is not a momentary injustice but a protracted one, with the harm accumulating over many years. The mean time to exoneration of nearly 12 years also suggests that current legal systems in Europe still struggle to identify and correct errors in a timely manner, and that structural barriers to post-conviction review remain significant across European jurisdictions.

### 3.3 More than 50% of the exonerations in homicide cases

The cases in EUREX are heavily concentrated in the most serious categories of violent crimes: with over half of all cases involving (attempted) murder or manslaughter (*n* = 81, 56.3%). Such findings are in line with what the Innocent Project (2026) and American research [59,60] have found. There are several reasons why more severe crimes are so prevalent in exoneration cases. First, serious crimes tend to involve more complex investigations, drawing on a wider range of forensic, testimonial, and expert evidence. The complexity of such investigations, particularly the reliance on forensic analysis, has been identified as a significant source of error in wrongful conviction cases [32]. Each additional layer of evidence introduces its own potential for mistakes, compounding the overall risk of a wrongful outcome. Second, serious crimes usually have higher sentences than less serious crimes, which makes it more likely for defendants to appeal a conviction [60]. Third, media coverage on high profile exoneration cases, such as murders, is usually higher than on low profile cases. Since the EUREX cases have been collected through almost exclusively publicly available sources, it may be that such high-profile media cases are easier to access and find compared to less severe crimes and their potential exonerations.

### 3.4 False confessions were the most prevalent contributing factor to wrongful convictions

False confessions emerged as the most prevalent contributing factor, present in 35.4% (*n* = 51) of the cases registered at EUREX. This stands in contrast to the NRE, where perjury or false accusation are present in most exoneration cases (64%) and false confessions only in 13% of the cases. This is surprising and may indicate that coercive interrogation tactics are not only applied in the United States but also in European countries. However, given that all founders of EUREX specialize in false confession research, they may have had increased exposure to relevant cases through their professional networks and prior engagement with the literature. The elevated prevalence of this factor should therefore be interpreted with this in mind. Nevertheless, these findings suggest that evidence-based reforms in suspect interviewing are needed across European jurisdictions, such as adopting the Méndez Principles [61].

Perjury or false accusations were the second most frequent contributing factor (*n* = 48, 33.3%) present in the current data. It is worth noting that this category encompasses both deliberate fabrications and unintentional false memories, and the EUREX coding does not yet distinguish between these two. This is a meaningful limitation: the policy implications differ considerably depending on whether false accusations arise from intentional deception, which might be addressed through stronger perjury sanctions or witness screening, or from memory distortion processes susceptible to suggestive interviewing, which call for procedural reforms in investigative interviewing practice. Future coding could shed light on those differences.

False or misleading forensic evidence was identified in 25.2% (*n* = 36) of EUREX cases. These numbers are similar to the NRE, where 29% of the cases listed this type of error. This convergence across two distinct legal contexts suggests that the vulnerability of criminal proceedings to forensic error is a systemic rather than jurisdiction-specific phenomenon. In one analysis of 732 NRE cases involving false or misleading forensic evidence five different error types were identified: misstatements in forensic science reports, classification errors, testimony errors, issues relating to trials and officers of the court, and evidence handling and reporting issues [32]. Often, errors occurred when forensic reports miscommunicated results, deviated from established standards, or omitted important limitations. The current EUREX data do not yet permit equivalent fine-grained differentiation between these subtypes, which limits the extent to which targeted remedial responses can be formulated. This is consequential because each mechanism implies a different remedial response: junk science calls for judicial gatekeeping and accreditation standards, while cognitive bias requires structural interventions such as blind verification procedures and case manager separation. Future in-depth coding of EUREX cases may allow for a more precise differentiation between error types of forensic evidence.

Flawed eyewitness identification (procedure) was present in 23.6% (*n* = 34) of the EUREX cases, closely aligning with the NRE, where this factor is reported in 27% of the cases. The documented variation in lineup procedures across European jurisdictions [62] means that the risk associated with this factor is not uniform across the dataset: exonerees from jurisdictions with less standardised identification procedures may be disproportionately represented. At the same time, as Mickes and Wixted [47] have recently argued, some eyewitness errors may be better understood as failures to preserve the integrity of initial identification responses rather than as fundamental limits of memory, a framing that shifts the locus of reform toward investigative procedure rather than eyewitness reliability per se.

Official misconduct was the least frequently coded contributing factor (*n* = 25, 17.4%), yet this figure almost certainly underestimates its true prevalence. Information about prosecutorial or police misconduct is often absent from public sources, and the EUREX coding procedure is therefore most likely to undercount this category. Findings on official misconduct should accordingly be interpreted with caution, and the relatively low frequency should not be read as evidence that misconduct is a minor contributor to European wrongful convictions. This gap itself is informative: it highlights a need for greater transparency in criminal justice proceedings and, where possible, for mechanisms such as conviction review units or independent oversight bodies that have access to case files beyond what is publicly available.

Across all five factors, it is important to note that contributing factors rarely operate in isolation. Co-occurrence of factors, for instance, a false confession obtained under conditions also involving official misconduct, reflects the systemic nature of wrongful convictions and cautions against single-factor explanations or reforms.

### 3.5 The real perpetrator was not found in 40% of the cases involving a crime

In the present dataset, the true perpetrator was identified in only 54 (59.3%) of the cases where a crime had happened, leaving 36 cases unresolved. This represents both an ongoing public safety risk and an unresolved injustice for the victims. Research from the United States found that among 109 identified actual perpetrators, 102 (approx. 94%) went on to commit further serious offences while the innocent person remained imprisoned [15].

### 3.6 Limitations

Data collection and interpretation of the registered exoneration cases in EUREX comes with several limitations. First, the EUREX dataset relies exclusively on publicly available sources, because we cannot access court files in most European jurisdictions. Therefore, exonerations cases involving serious crimes are substantially overrepresented, as these are more likely to be covered in the media. Furthermore, certain contributing factors, most notably official misconduct, and other detailed case information are systematically harder to detect through public sources compared to case files and the information collected is more prone to errors. Second, language barriers present a practical barrier. Encountering case information in numerous languages restricted the identification of eligible cases and the accuracy of coding. However, every effort was made to ensure accurate translation and interpretation of case information with the help of volunteers and local innocence project partners. Third, not all European countries are currently represented, primarily due to the lack of native-speaker volunteers or contacts in those countries. In addition, even in countries that have been examined, coverage remains incomplete. In some jurisdictions, relevant cases are anonymized or described only in limited detail in publicly available sources, which makes verification and coding challenging. In other instances, cases identified in prior research cannot yet be included because available information is insufficient to meet EUREX's inclusion criteria (e.g., lack of detail required for coding or inability to assign a pseudonym). As a result, some known cases are currently being re-examined in collaboration with local researchers and may be incorporated into the registry in future updates. Fourth, by only collecting exoneration cases in which the original conviction occurred in 1970 or later, the registry excludes many earlier cases. This temporal restriction was applied to focus on cases within a more consistent modern legal framework following key legal reforms. Nevertheless, this choice means that historical wrongful convictions are not represented in the current dataset. For example, De Beuf and Otgaar [63] identified 28 exoneration cases in Belgium between 1830 and 1963. Finally, the definitional framework for wrongful convictions adopted by EUREX means that a substantial number of cases are excluded from the registry by design (e.g., cases in which individuals were acquitted on appeal before a final conviction). This narrow definition was necessary to ensure cross-national comparability across diverse European legal systems, where the legal routes to exoneration vary considerably. In addition to these definitional exclusions, some wrongful convictions are likely not yet captured due to incomplete coverage across jurisdictions and limited access to case information. While the chosen framework ensures comparability, it results in an undercount of the true scope of wrongful conviction in Europe. Future research might consider developing complementary registries or coding schemes that capture a broader category of miscarriages of justice.

### 3.7 Implications and future directions

Beyond these methodological limitations, the EUREX data point to several concrete directions for future research and policy. The near-total absence of formal case review units in the cases documented here, present in only 8.3% of cases, raises important questions about how wrongful convictions are identified and corrected across Europe in the absence of dedicated oversight mechanisms. Comparative research examining the structure, mandate, and effectiveness of existing review bodies, such as the Criminal Cases Review Commissions in the UK or Norway, could inform efforts to establish equivalent institutions in jurisdictions where none currently exist [9].

Furthermore, the high prevalence of false confessions within the dataset suggests that coercive and confession-oriented interrogation practices are still frequently employed in European contexts. Historically, such false confessions have served as a catalyst for reform in some European countries. For instance, in England and Wales, wrongful convictions due to false confessions led to significant legal reforms in 1984, followed by a growing body of research that contributed to the development of fair and empirically grounded interviewing practices [64]. Building on these developments, the PEACE model was introduced in the United Kingdom in the 1990s, marking the first systematic implementation of the investigative interviewing approach. This approach emphasizes information gathering over confession seeking and was subsequently adopted by other countries—often in response to false confessions—such as Norway (e.g., [65]). More recently, the Méndez Principles have further reinforced this paradigm by promoting non-coercive, rapport-based, and evidence-driven interviewing practices [61]). These principles provide a guideline for other European countries to implement such interviewing practices to prevent wrongful convictions based on false confessions.

### 3.8 Conclusion

Taken together, the EUREX dataset offers a valuable, though incomplete, empirical basis for examining wrongful convictions in Europe. Despite clear limitations in coverage and data availability, the observed patterns—namely the increase in recorded exonerations over time, substantial delays between conviction and exoneration, the predominance of serious violent offences, and the co-occurrence of multiple contributing factors—closely align with international findings. This convergence suggests that wrongful convictions are not isolated anomalies but reflect recurring, systemic vulnerabilities within criminal justice processes. At the same time, the analysis highlights distinct structural challenges in the European context, particularly limited access to case materials and the relative absence of specialized post-conviction review units.

During the preparation of this work the authors used ChatGPT (OpenAI), Claude (Anthropic), and Copilot (Microsoft) in order to support background research and writing assistance. After using these tools, the authors reviewed and edited the content as needed and take full responsibility for the content of the published article.

## CRediT authorship contribution statement

**Teresa Schneider:** Conceptualization, Project administration, Writing – original draft, Writing – review & editing. **Linda M. Geven:** Conceptualization, Formal analysis, Project administration, Writing – review & editing. **Jennifer M. Schell-Leugers:** Conceptualization, Formal analysis, Project administration, Writing – original draft, Writing – review & editing.

## Declaration of competing interest

The authors declare that they have no known competing financial interests or personal relationships that could have appeared to influence the work reported in this paper.

## Footnotes

1 https://innocenceproject.org/dna-exonerations-in-the-united-states/.

2 https://exonerationregistry.org/.

3 https://evidencebasedjustice.exeter.ac.uk/miscarriages-of-justice-registry/.

4 https://www.wrongfulconvictions.ca.

5 https://registryofexonerations.eu.

6 https://innocenceproject.org/misapplication-of-forensic-science/.

7 https://innocenceproject.org/eyewitness-misidentification/.

8 We selected 1970 as the starting point in order to align with the methodology of the Evidence-Based Justice Lab. This choice is further justified by the extensive legal and judicial transformations that occurred across many European countries during the late 1960s and 1970s, which marked an important shift in criminal justice systems.
